# The use of complementary and alternative medicine by 7427 Australian women with cyclic perimenstrual pain and discomfort: a cross-sectional study

**DOI:** 10.1186/s12906-016-1119-8

**Published:** 2016-05-18

**Authors:** Carole Fisher, Jon Adams, Louise Hickman, David Sibbritt

**Affiliations:** Australian Research Centre in Complementary and Integrative Medicine (ARCCIM), Faculty of Health, University of Technology Sydney, Level 8, Building 10, 2353 Jones St, Sydney, NSW 2007 Australia

**Keywords:** Endometriosis, Premenstrual syndrome, Irregular periods, Heavy periods, Severe dysmenorrhoea, Complementary and Alternative medicine

## Abstract

**Background:**

To assess the prevalence of cyclic perimenstrual pain and discomfort and to detail the pattern of complementary and alternative (CAM) use adopted by women for the treatment of these symptoms.

**Methods:**

Data from the 2012 national Australian Longitudinal Study of Women’s Health (ALSWH) cross-sectional survey of 7427 women aged 34–39 years were analysed to estimate the prevalence of endometriosis, premenstrual syndrome (PMS), irregular or heavy periods and severe dysmenorrhoea and to examine the association between their symptoms and their visits to CAM practitioners as well as their use of CAM therapies and products in the previous 12 months.

**Results:**

The prevalence of endometriosis was 3.7 % and of the perimenstrual symptoms assessed, PMS was most prevalent at 41.2 % whilst irregular bleeding (22.2 %), heavy periods (29.8 %) and severe period pain (24.1 %) were reported at lower levels. Women with endometriosis were more likely than non-sufferers to have consulted with a massage therapist or acupuncturist and to have used vitamins/minerals, yoga/meditation or Chinese medicines (*p* < 0.05). PMS sufferers were more likely to consult with an osteopath, massage therapist, naturopath/herbalist or alternative health practitioner and to have used all forms of CAM therapies except Chinese medicines than women who had infrequent PMS (all *p* < 0.05). Women with irregular periods did not have different patterns of CAM use from non-sufferers and those with heavy periods did not favour any form of CAM but were less likely to visit a massage therapist or use yoga/meditation than non-sufferers (*p* < 0.05). For women with severe dysmenorrhoea there was no difference in their visits to CAM practitioners compared to non-sufferers but they were more likely to use aromatherapy oils (*p* < 0.05) and for more frequent dysmenorrhoea also herbal medicines, Chinese medicines and other alternative therapies compared to non-sufferers (all *p* < 0.05).

**Conclusions:**

There is a high prevalence of cyclic perimenstrual pain and discomfort amongst women in this age group. Women were using CAM differentially when they had specific symptoms of cyclic perimenstrual pain and discomfort. The use of CAM needs to be properly assessed to ensure their safe, effective use and to ascertain their significance as a treatment option enabling women with menstrual problems and their care providers to improve their quality of life.

## Background

Menstruation is a normal, cyclic event spanning a women’s life from the onset of puberty (usually around 12–13 years of age) through to menopause (which most women experience around 50 years of age). Although the phenomenon of cyclic problems experienced by women during their reproductive years had been recognised by Hippocrates [1] modern science has not yet fully explained the causes of the variety of symptoms that can accompany the menstrual cycle [1–4]. Moreover symptoms have been treated as separate entities, most commonly identified as either premenstrual syndrome (PMS) or dysmenorrhoea, although their co-existence was highlighted by Bancroft in 1995 [2]. Affective symptoms like irritability and depression and physical symptoms such as bloating, dysmenorrhoea, nausea and breast tenderness often occur in the luteal phase of the cycle and/or into menstruation. Symptoms may vary in severity and scope from one cycle to the next and are likely due to a number of different factors [2, 5–7]. Such changes were labelled ‘cyclic perimenstrual pain and discomfort’ (CPPD) by the Association of Women’s Health, Obstetric and Neonatal Nurses [5] to better reflect the protean nature of symptoms. It is estimated that between 80 and 97 % of women worldwide and across age groups experience at least one symptom during their reproductive life [6–9]. For up to 40 % of women [7] symptoms are moderate and for a further 2–10 % of women symptoms are severe enough to interfere with normal daily life [4, 6, 8, 10]. It is possible that the prevalence of CPPD has increased as modern women are exposed to sex-hormone cycles for a greater proportion of their lives due to earlier onset of menarche, reduced number of births and the delayed resumption of menstrual cycles consequent on breastfeeding. Though cultural differences may change women’s perception of, and treatment-seeking behaviour for, CPPD, its prevalence does not appear to be a cultural factor [11–14].

Complementary and alternative medicine (CAM) includes a range of diverse health-related strategies that can be described as predominantly operating outside the conventional medical curriculum and medical profession [15]. The prevalence of CAM use worldwide is substantial [16, 17], especially amongst women [18–22]. There is an acceptance of a multi-factorial aetiology for CPPD [2, 4, 23] and to-date conventional treatment protocols, which focus on symptom-relief, ranging from counselling to pharmaceutical to surgical, have been employed. Neither single nor combinations of conventional therapies have produced consistent positive outcomes for CPPD [5] and a large variety of both conventional and CAM approaches have been promoted by medical practitioners [5, 24]. Indeed there are clinical trials that support the use of CAM for aspects of CPPD such as *Vitex agnus-castus* [25] or Chinese herbs [26] for PMS and Transcutaneous Electrical Nerve Stimulation (TENS) [27] or acupuncture [28] for dysmenorrhoea. Previous work exploring CAM use for CPPD symptoms has revealed prevalence rates between 3 and 70 % [12, 13, 29–44]. although prevalence of women’s CAM use at any point for CPPD is likely to be very much higher [45–47]. Unfortunately, the extent of this behaviour is unknown, particularly in Western countries as few recent studies have been undertaken. In addition there is a lack of good quality studies published in the peer-reviewed literature and surveys have varied widely in sample size and source, baseline and timeframe for measurement, questionnaire quality and method of data collection. All health practitioners need to be better-informed about this usage to enable more effective and safer symptom management.

Therefore, in an attempt to fill this gap in knowledge regarding CAM use for CPPD, this study presents a detailed analysis of the prevalence of CPPD symptoms in women from the large, nationally-representative Australian Longitudinal Study on Women’s Health (ALSWH), examining the specific CAM adopted by women, over a twelve month timeframe, according to their symptoms.

## Method

### Sample

Data was obtained from the (born in) 1973–78 cohort of the Australian Longitudinal Study on Women’s Health (ALSWH). In 1996, the ALSWH participants were randomly selected from the national Medicare database, which is the universal healthcare provision for all Australians. The recruited sample comprised over 58,000 women from 3 aged groups (ie. ‘young’: 18–23 years; ‘mid age’: 45–50 years; ‘older’: 70–75 years), to examine women in the key stages of the lifespan. The recruited women have been surveyed, via postal questionnaires, at regular 3-yearly intervals. The ALSWH was designed to follow the cohorts over 20 years to monitor changes in health and are intended to help guide national health policy and provision. The analyses presented in this study were restricted to Survey 6 (conducted in 2012) of the young cohort (when they were aged 34–39 years), which included 8009 respondents, a retention rate of eligible participants for this survey of 61.6 %. In the first survey 14,247 women in this age group participated, census data for this demographic at the time of recruitment was 759,680. Ethical approval for the ALSWH was gained from the Human Ethics Committees at the University of Queensland and University of Newcastle. The study participants provided written consent.

### Cyclic perimenstrual pain and discomfort symptoms

Women were asked if they had been diagnosed with endometriosis in the last 3 years. In addition, they were also asked how frequently they experienced premenstrual tension, irregular periods, heavy periods and severe period pain in the previous 12 months, with the response option being ‘never’, ‘rarely’, ‘sometimes’ or ‘often’.

### Complementary and alternative medicine use

Women’s consultations with CAM practitioners were ascertained by questionnaire items asking them is they had consulted any of a list of practitioners, for their own health, in the previous 12 months. The list of CAM practitioners included: chiropractor, osteopath, massage therapist, acupuncturist, naturopath/herbalist, and ‘another alternative’ health practitioner.

Women’s use of CAM practices or products was ascertained by questionnaire items asking them how frequently they had used any of a list of therapies or products, for their own health, in the previous 12 months. The list of therapies or products included: vitamins/minerals, yoga/meditation, herbal medicines, aromatherapy oils, Chinese medicine and ‘other alternative practices or products’. Possible response options were ‘never’, ‘rarely’, ‘sometimes’ and ‘often’. Those responding ‘never’ or ‘rarely’ were categorised as non-users and the ‘sometimes’ and ‘often’ responders were classified as users for analysis purposes.

### Confounders

Potential confounders identified (that were available in the questionnaire) were the demographic factors area of residence, educational status, ability to manage on income and marital status and the co-morbidities of insulin-dependent (Type 1) diabetes, non-insulin dependent (Type 2) diabetes, low iron (iron deficiency or anaemia), depression, anxiety disorder, asthma, ‘other cancer’ and hypertension.

Area of residence was categorised as either urban or rural. Educational status was grouped as one of three categories: no formal qualifications, year 10 or equivalent (eg. school certificate), year 12 or equivalent (e.g. higher school certificate); trade/apprenticeship or certificate/diploma; and university degree. Ability to manage on available income was also grouped as one of three categories: it is impossible or it is difficult all of the time; it is difficult some of the time; and it is not too bad or it is easy. Marital status was grouped into three categories: never married; married/de facto; and separated or divorced or widowed.

### Statistical analysis

Bivariate analyses testing the association between CPPD symptom and CAM practitioner or CAM therapy use was conducted using chi-square tests. Logistic regression models were used to determine magnitude of association between CPPD symptom and CAM practitioner or therapy use, with adjustment for confounding variables. Statistical significance was set at the α = 0.05 level for all analyses, using the statistical package STATA 14.0.

## Results

There were 7427 women who indicated they had not had a bilateral oophorectomy and were either not pregnant or unsure if they were pregnant. Amongst these women, the prevalence of CPPD-related problems was 3.7 % for endometriosis, 41.2 % had suffered from PMS sometimes or often, 22.2 % had had irregular periods sometimes or often, 29.8 % had experienced heavy periods sometimes or often and 24.1 % had had severe period pain sometimes or often. Those women with endometriosis may account for around 4.9 % of more frequent (ie. sometimes/often) PMS sufferers, 6.1 % of more frequent cases of irregular periods, 6.1 % of more frequent heavy periods and 8.3 % of more frequent severe period pain sufferers.

The association between CAM practitioner consultations and CPPD symptoms are presented in Table 1. Women with endometriosis were significantly more likely to consult with a massage therapist, acupuncturist, and/or naturopath/herbalist compared to women who did not have endometriosis (all *p* < 0.05). Consultations with a naturopath/herbalist were significantly higher for women suffering with irregular or heavy periods, compared to those without irregular and/or heavy periods (all *p* < 0.05). Similarly, women with severe period pain were significantly more likely to consult with an acupuncturist and/or a naturopath/herbalist, compared to women without severe period pain (all *p* < 0.05). Compared to women without PMS, women with PMS were significantly more likely to consult with a massage therapist, acupuncturist, naturopath/herbalist and ‘other alternative health practitioner’ (all *p* < 0.05).

**Table 1:** The association between cyclic perimenstrual pain and discomfort (CPPD) and consultations with complementary and alternative medicine practitioners

Cyclic perimenstrual pain and discomfort symptoms	Chiropractor		Osteopath		Massage Therapist		Acupuncturist		Naturopath/Herbalist		Other CAM Practitioner	
	Yes (*n* = 1480)	No (*n* = 5929)	Yes (*n* = 621)	No (*n* = 6781)	Yes (*n* = 3152)	No (*n* = 4260)	Yes (*n* = 667)	No (*n* = 6733)	Yes (*n* = 835)	No (*n* = 6567)	Yes (*n* = 568)	No (*n* = 6833)
	%	%	%	%	%	%	%	%	%	%	%	%
Endometriosis ^C,D,E^												
No Yes	95 5	96 4	96 4	96 4	95 5	97 3	93 7	97 3	94 6	97 3	95 5	96 4
PMS ^C, D, E, F^												
Never Rarely Sometimes Often	36 22 29 13	38 21 28 13	33 22 28 17	38 21 28 13	34 22 30 14	40 21 27 12	31 21 31 17	38 21 28 13	29 19 31 21	39 21 28 12	29 22 32 17	38 21 28 13
Irregular Periods ^E^												
Never Rarely Sometimes Often	62 14 15 9	61 17 13 9	64 16 12 8	61 16 14 9	61 16 14 9	61 17 13 9	58 17 14 11	62 16 13 9	56 17 16 11	62 16 13 9	57 16 16 11	62 16 13 9
Heavy Periods ^E^												
Never Rarely Sometimes Often	51 19 18 12	53 17 19 11	54 16 19 11	52 18 19 11	52 18 19 11	53 17 18 12	52 17 19 12	53 18 18 11	46 18 21 15	53 18 18 11	49 20 17 14	53 17 19 11
Painful Periods ^D,E^												
Never Rarely Sometimes Often	53 24 15 8	53 22 17 8	52 22 17 9	53 23 16 8	51 24 17 8	54 22 16 8	48 23 18 11	54 22 16 8	48 24 15 13	54 23 16 7	48 24 18 10	53 23 16 8

^A^statistically significant association with chiropractor

^B^statistically significant association with osteopath

^C^statistically significant association with massage therapist

^D^statistically significant association with acupuncturist

^E^statistically significant association with naturopath/herbalist

^F^statistically significant association with ‘other CAM’ practitioner

Table 2 shows associations between use of CAM practices/products and CPPD symptoms. Women with PMS and/or painful periods were significantly more likely to use all CAM practices/products frequently, compared to women without PMS and/or painful periods (all *p* < 0.05). With the exception of aromatherapy oils, endometriosis sufferers were significantly more likely to use all other CAM practices/products, compared to women without endometriosis (all *p* < 0.05). Women who were suffering with irregular periods were more likely to use vitamins/minerals, herbal medicines, Chinese medicines, and/or ‘other alternative practices and products’, compared to women who did not suffer with irregular periods (all *p* < 0.05). Women with menorrhagia were significantly more likely to use herbal medicines, aromatherapy oils and/or ‘other alternative practices and products’ compared to non-sufferers (all *p* < 0.05).

**Table 2:** The association between cyclic perimenstrual pain and discomfort (CPPD) and use of complementary and alternative medicine practices and products

Cyclic perimenstrual pain and discomfort symptoms	Vitamins/Minerals		Yoga/Meditation		Herbal medicines		Aromatherapy oils		Chinese medicines		Other alternative therapies	
	Never or Rarely (*n* = 2377)	Sometimes or often (*n* = 5043)	Never or Rarely (*n* = 5705)	Sometimes or often (*n* = 1714)	Never or Rarely (*n* = 5917)	Sometimes or often (*n* = 1500)	Never or Rarely (*n* = 6212)	Sometimes or often (*n* = 1203)	Never or Rarely (*n* = 7016)	Sometimes or often (*n* = 403)	Never or Rarely (*n* = 6659)	Sometimes or often (*n* = 741)
	%	%	%	%	%	%	%	%	%	%	%	%
Endometriosis ^A,B,C,E,F^												
No Yes	98 2	96 4	97 3	94 6	97 3	95 5	96 4	96 4	97 3	92 8	97 3	94 6
PMS ^A,B,C,D,E,F^												
Never Rarely Sometimes Often	41 22 26 11	36 20 30 14	39 21 28. 12	32 20 31 17	40 21 27 12	29 20 33 18	39 21 28 12	29 20 33 18	38 21 28 13	31 20 31 18	38 22 28 12	28 18 34 20
Irregular Periods ^A,C,E,F^												
Never Rarely Sometimes Often	64 16 12 8	60 17 14 9	61 17 13 9	60 16 15 9	62 16 13 9	57 17 16 10	62 16 13 9	58 18 14 10	62 16 13 9	55 16 17 12	62 16 13 9	56 16 16 12
Heavy Periods ^C,D,F^												
Never Rarely Sometimes Often	55 17 17 11	51 18 19 12	53 17 18 12	51 18 19 12	54 17 18 11	47 18 20 15	54 17 18 11	47 18 21 14	53 17 19 11	49 17 18 16	54 17 18 11	45 19 20 16
Painful Periods ^A,B,C,D,E,F^												
Never Rarely Sometimes Often	55 23 15 7	52 22 17 9	54 23 16 7	50 23 17 10	55 22 16 7	45 24 19 12	55 23 15 7	45 22 21 12	53 23 16 8	46 23 17 14	54 23 16 7	43 24 19 14

^A^statistically significant association with vitamins/minerals ^D^ statistically significant association with aromatherapy oils

^B^statistically significant association with yoga or meditation ^E^ statistically significant association Chinese medicines

^C^statistically significant association with herbal medicines ^F^ statistically significant association with ‘other alternative therapies’

Logistic regression modelling was used to determine the magnitude of associations between CPPD related problems and consultations with CAM practitioners, after adjusting for confounders (Table 3). Endometriosis sufferers were nearly 50 % more likely to have consulted a massage therapist (OR = 1.48; 95 % CI: 1.14, 1.92) and nearly twice as likely to have consulted with an acupuncturist (OR = 1.79; 95 % CI: 1.26, 2.56), compared to women without endometriosis. Women who ‘sometimes’ suffered PMS were more likely to have visited with a massage therapist (OR = 1.29; 95 % CI: 1.12, 1.48) but for ‘often’ sufferers this association was higher (OR = 1.47, 95 % CI: 1.23, 1.76), whilst ‘sometimes’ sufferers were also more likely to visit a naturopath/herbalists (OR = 1.48, 95 % CI: 1.19, 1.85) but were more than twice as likely to have done so when the PMS occurred ‘often’ (OR = 2.12, 95 % CI: 1.62, 2.76). Those women who indicated PMS occurred ‘often’ were over 60 % more likely to have had osteopathic treatment (OR = 1.64, 95 % CI: 1.19, 2.20), and/or visited ‘another alternative health practitioner’ (OR = 1.66, 95 % CI: 1.20–2.29). Women who experienced heavy periods ‘often’ were 30 % (OR = 0.70, 95 % CI: 0.56, 0.87) less likely to have used a massage therapist. No statistically significant associations were observed between any of the CAM practitioner groups and irregular periods or painful periods.

**Table 3:** The odds ratio for association of cyclic perimenstrual pain and discomfort and consultations with complementary and alternative medicine practitioners

Cyclic Perimenstrual Pain and Discomfort Symptom	Chiropractor (*n* = 7005)	Osteopath (*n* = 6997)	Massage Therapist (*n* = 7008)	Acupuncturist (*n* = 6999)	Naturopath/Herbalist (*n* = 6999)	Other CAM Practitioner (*n* = 6996)
	O/R (C.I.)	O/R (C.I.)	O/R (C.I.)	O/R (C.I.)	O/R (C.I.)	O/R (C.I.)
Endometriosis^C,D^						
No Yes	1.00 1.30 (0.96–1.76)	1.00 1.11 (0.71–1.73)	1.00 1.48 (1.14–1.92)	1.00 1.79 (1.26–2.56)	1.00 1.32 (0.92–1.89)	1.00 1.21 (0.78–1.88)
PMS						
Never Rarely Sometimes^C,E^ Often^B,C,E,F^	1.00 1.15 (0.97–1.37) 1.12 (0.94–1.33) 1.06 (0.85–1.33)	1.00 1.27 (0.99–1.62) 1.26 (0.98–1.61) 1.64 (1.19–2.20)	1.00 1.20 (1.05–1.38) 1.29 (1.12–1.48) 1.47 (1.23–1.76)	1.00 1.23 (0.96–1.58) 1.32 (1.04–1.68) 1.47 (1.09–1.98)	1.00 1.24 (0.99–1.57) 1.48 (1.19–1.85) 2.12 (1.62–2.76)	1.00 1.40 (1.08–1.83) 1.45 (1.12–1.89) 1.66 (1.20–2.29)
Irregular Periods						
Never Rarely Sometimes Often	1.00 0.80 (0.67–0.97) 1.08 (0.90–1.30) 0.91 (0.72–1.14)	1.00 0.93 (0.72–1.20) 0.82 (0.61–1.09) 0.89 (0.64–1.24)	1.00 0.86 (0.74–0.99) 1.13 (0.97–1.32) 1.00 (0.83–1.20)	1.00 1.08 (0.84–1.38) 1.01 (0.77–1.32) 1.15 (0.85–1.56)	1.00 1.02 (0.82–1.28) 1.20 (0.95–1.51) 1.15 (0.88–1.52)	1.00 0.88 (0.67–1.16) 1.14 (0.87–1.50) 1.07 (0.77–1.48)
Heavy Periods						
Never Rarely Sometimes Often^C^	1.00 1.21 (1.00–1.46) 0.99 (0.81–1.22) 1.13 (0.87–1.47)	1.00 0.78 (0.59–1.03) 0.78 (0.58–1.03) 0.58 (0.40–0.86)	1.00 0.96 (0.83–1.12) 0.84 (0.71–0.99) 0.70 (0.56–0.87)	1.00 0.81 (0.62–1.06) 0.77 (0.58–1.01) 0.60 (0.42–.087)	1.00 1.06 (0.83–1.35) 1.11 (0.86–1.42) 0.97 (0.70–1.34)	1.00 1.03 (0.78–1.36) 0.77 (0.57–1.05) 0.89 (0.60–1.30)
Painful Periods						
Never Rarely Sometimes Often	1.00 0.96 (0.81–1.15) 0 .86 (0.69–1.06) 0 .96 (0.71–1.29)	1.00 1.11 (0.86–1.43) 1.25 (0.92–1.69) 1.48 (0.98–2.23)	1.00 1.16 (1.01–1.34) 1.06 (0.89–1.27) 1.14 (0.89–1.46)	1.00 1.15 (0.90–1.47) 1.20 (0.89–1.60) 1.70 (1.16–2.51)	1.00 0.94 (0.75–1.18) 0.69 (0.52–0.90) 1.11 (0.78–1.57)	1.00 1.04 (0.80–1.36) 1.05 (0.77–1.44) 1.09 (0.71–1.68)

^A^statistically significant association with chiropractor

^B^statistically significant association with osteopath

^C^statistically significant association with massage therapist

^D^statistically significant association with acupuncturist

^E^statistically significant association with naturopath/herbalist

^F^statistically significant association with ‘other CAM’ practitioner

Adjusted for confounding variables - marital status, area of residence, educational status, low iron, depression and anxiety disorder

Table 4 shows the results of logistic regression modelling used to determine the magnitude of associations between CPPD related problems and use of CAM practices/products, after adjusting for confounders. Women with endometriosis were more likely to have used vitamins/minerals (OR = 1.72; 95 % CI: 1.24, 2.38), yoga/meditation (OR = 1.80; 95 % CI: 1.37, 2.38) and/or Chinese medicines (OR = 1.86; 95 % CI: 1.22, 2.83), compared to women without endometriosis. PMS sufferers showed around a 30 % (OR = 1.31; 95 % CI: 1.09, 1.56) increased likelihood to have used herbal medicine if their symptoms were ‘rare’ but this likelihood increased for ‘sometimes’ sufferers (OR = 1.49; 95 % CI: 1.25, 1.77) and was greatest for ‘often’ sufferers (OR = 1.72; 95 % CI: 1.39, 2.14). Those who suffered PMS ‘sometimes’ or ‘often’ were also more likely to use vitamins/minerals (OR = 1.31; 95 % CI: 1.13, 1.52 and OR = 1.47; 95 % CI: 1.21, 1.80 respectively), yoga/meditation (OR = 1.34; 95 % CI: 1.14, 1.59 and OR = 1.64; 95 % CI: 1.33, 2.02 respectively), aromatherapy oils (OR = 1.49; 95 % CI: 1.23, 1.80 and OR = 1.53; 95 % CI: 1.21, 1.94 respectively) and/or ‘other alternative therapies’ (OR = 1.46; 95 % CI: 1.16, 1.84 and OR = 1.69; 95 % CI: 1.27, 2.23 respectively). Women who experienced heavy periods ‘often’ were less likely to use yoga/meditation (OR = 0.68; 95 % CI: 0.53, 0.88), compared to women who ‘never’ experienced heavy periods. Women who ‘sometimes’ had severe period pain were more likely to have used aromatherapy oils (OR = 1.46; 95 % CI: 1.17, 1.82) but if the dysmenorrhoea was ‘often’ this likely use increased to over 70 % (OR = 1.76; 95 % CI: 1.30, 2.38). This group of ‘often ‘dysmenorrhoea sufferers were also more likely to have used herbal medicines (OR = 1.63; 95 % CI: 1.24, 2.15) and/or ‘other alternative therapies’ (OR = 1.73; 95 % CI: 1.21, 2.47), compared to women who ‘never’ experienced dysmenorrhoea. There were no significant associations between the irregular period categories and use of any CAM therapies or products.

**Table 4:** The odds ratio for association between cyclic perimenstrual pain and discomfort and use of complementary and alternative medicine practices and products

Cyclic Perimenstrual Pain and Discomfort Symptom	Vitamins/minerals (*n* = 7017)	Yoga/meditation (*n* = 7014)	Herbal medicines (*n* = 7013)	Aromatherapy (*n* = 7012)	Chinese medicines (*n* = 7015)	Other alternative therapies (*n* = 6996)
	O/R (C.I.)	O/R (C.I.)	O/R (C.I.)	O/R (C.I.)	O/R (C.I.)	O/R (C.I.)
Endometriosis^A,B,E^						
No Yes	1.00 1.72 (1.24–2.38)	1.00 1.80 (1.37–2.38)	1.00 1.34 (1.00–1.79)	1.00 0.86 (0.61–1.22)	1.00 1.86 (1.22–2.83)	1.00 1.28 (0.88–1.85)
PMS						
Never Rarely^C^ Sometimes^A,B,C,D,F^ Often^A,B,C,D,F^	1.00 1.08 (0.93–1.25) 1.31 (1.13–1.52) 1.47 (1.21–1.80)	1.00 1.20 (1.01–1.41) 1.34 (1.14–1.59) 1.64 (1.33–2.02)	1.00 1.31 (1.09–1.56) 1.49 (1.25–1.77) 1.72 (1.39–2.14)	1.00 1.27 (1.04–1.54) 1.49 (1.23–1.80) 1.53 (1.21–1.94)	1.00 1.15 (0.84–1.57) 1.24 (0.91–1.68) 1.38 (0.95–2.01)	1.00 1.14 (0.89–1.46) 1.46 (1.16–1.84) 1.69 (1.27–2.23)
Irregular Periods						
Never Rarely Sometimes Often	1.00 1.16 (0.99–1.36) 1.21 (1.02–1.44) 1.13 (0.93–1.38)	1.00 0.94 (0.79–1.12) 1.10 (0.92–1.32) 0.96 (0.77–1.20)	1.00 0.97 (0.81–1.16) 1.15 (0.96–1.38) 1.00 (0.80–1.24)	1.00 1.02 (0.84–1.23) 0.96 (0.78–1.18) 0.91 (0.72–1.17)	1.00 1.06 (0.77–1.46) 1.43 (1.04–1.96) 1.34 (0.93–1.94)	1.00 0.89 (0.70–1.13) 1.11 (0.87–1.41) 1.09 (0.82–1.45)
Heavy Periods						
Never Rarely Sometimes Often^B^	1.00 1.04 (0.89–1.23) 0.97 (0.82–1.16) 0.78 (0.62–0.98)	1.00 0.97 (0.81–1.16) 0.90 (0.74–1.09) 0.68 (0.53–0.88)	1.00 0.92 (0.76–1.12) 0.93 (0.76–1.13) 0.92 (0.72–1.19)	1.00 0.92 (0.75–1.14) 0.94 (0.76–1.16) 0.82 (0.62–1.08)	1.00 0.83 (0.59–1.16) 0.65 (0.46–0.94) 0.72 (0.46–1.11)	1.00 1.04 (0.81–1.35) 0.89 (0.69–1.16) 0.85 (0.61–1.19)
Painful Periods						
Never Rarely Sometimes^D^ Often^C,D,F^	1.00 0.85 (0.73–0.98) 0.94 (0.78–1.13) 1.15 (0.88–1.50)	1.00 1.01 (0.86–1.20) 1.04 (0.85–1.28) 1.31 (0.99–1.73)	1.00 1.10 (0.92–1.31) 1.17 (0.95–1.44) 1.63 (1.24–2.15)	1.00 1.04 (0.86–1.27) 1.46 (1.17–1.82) 1.76 (1.30–2.38)	1.00 1.18 (0.87–1.62) 1.21 (0.83–1.76) 1.79 (1.11–2.87)	1.00 1.16 (0.91–1.47) 1.21 (0.92–1.60) 1.73 (1.21–2.47)

^A^statistically significant association with vitamins/minerals

^B^statistically significant association with yoga or meditation ^E^ statistically significant association Chinese medicines

^C^statistically significant association with herbal medicines ^F^ statistically significant association with ‘other alternative therapies’

^D^statistically significant association with aromatherapy oils

Adjusted for confounding variables - marital status, area of residence, educational status, low iron, depression and anxiety disorder

## Discussion

Results from this analysis of 34–39 year old menstruating women derived from a large nationally representative sample of Australian women contributes important information regarding the prevalence of CPPD symptoms and their relationship to the differential adoption of CAM. The analysis further indicates that women experiencing CPPD symptoms are likely to be using CAM of which the majority involves CAM products and therapies rather than consultations with CAM practitioners.

### Prevalence of CPPD

The prevalence for endometriosis in this cohort was 3.7 %, which is supported by the Global Burden of Disease Study 2013 which estimated the prevalence of endometriosis at 4.8 % for the years 2006–13 [48].

The prevalence for PMS of 43.3 % in our study compares favourably with that from international data of 47.8 % from a meta-analysis based on 17 international studies from 1996 to 2011 [49]. Irregular periods were experienced by 22.3 % of women in our cohort. A broad range of prevalence estimates of irregular periods 6.5–83.3 % was reported from a systematic review of data from developing countries [50] and 25.6 % of 18–40 year-old nulliparous Danish women self-reported irregular periods [51]. Our cohort had a menorrhagia prevalence of 29.9 % which tallies well with self-reported assessments elsewhere; a review of the literature up to 2005 found six reports of prevalence of heavy periods of between 10 and 30 % with lower levels objectively determined while higher levels were based upon subjective assessments [52]. The prevalence of severe period pain determined from our data, at 24.2 %, falls within the range derived from a 2002 to 2011 review across 15 studies of 2–29 % severe period pain [9]. Comparative data for the same age group is limited, but includes Korean [35], Japanese [29] and UK [53] surveys where the prevalence was 68, 29.2 and 15 % respectively however only the latter two were based on severe levels of dysmenorrhoea.

### CAM use for CPPD

After adjusting for potential confounders, women with endometriosis in our sample were much more likely to visit with a massage therapist and/or acupuncturist and to use vitamins/minerals, yoga/meditation and/or Chinese medicines. Massage and acupuncture has previously been reported by endometriosis sufferers as satisfactory treatment for its associated leg pain [41] and there is evidence that Chinese medicines and acupuncture can reduce both endometriosis signs and symptoms. [54] Whilst no other direct research has been carried out into CAM for endometriosis there are studies indicating vitamins/minerals for reducing dysmenorrhoea which is a significant factor in symptomatic endometriosis [55].

Our analyses indicate that frequent PMS sufferers are more likely than those women who never or rarely experience this symptom to visit with a massage therapist or naturopath/herbalist, as well as increasing the likelihood of visiting an osteopath. All CAM practices and products included in this study were used with a significantly greater likelihood by women with PMS than those who either did not, or rarely experienced PMS, except for Chinese medicines and that there was an increasing trend to use herbal medicine with increasing frequency of PMS. PMS is the most common CPPD symptom in our cohort and lack of specific, effective medications may account for the higher observed likelihood of use of multiple CAM practitioners and therapies. As reported in previous studies, more than half of PMS sufferers who used vitamins/dietary supplements [46, 56, 57], acupuncture [46, 58], homeopathy [46, 59], yoga/mind body [57, 59] and massage [56, 57] have reported finding them satisfactory as a treatment. The association between CAM practitioner visits in our study provides some of the only data available with regard to PMS sufferers. A review of evidence for CAM and PMS highlighted at least a 50 % improvement in symptoms from studies of women using either acupuncture or herbal medicine (both Western and Chinese) [60]. This is in contrast to our data which found no association between more frequent levels of PMS and visits to an acupuncturist or use of Chinese medicines and this may reflect the cultural differences in behaviour of Australian women with less exposure and knowledge of traditional Chinese medicine than women in cultures where it is more main stream. Systematic reviews of PMS treatment have indicated CAM that may be useful includes massage therapy, reflexology, calcium, vitamin B_6_ [61, 62] and possibly magnesium and yoga [62]. Our data indicates that women with PMS are indeed adopting these CAM.

Irregular and heavy periods and CAM use has not been well investigated, although there are qualitative studies showing that either type of irregular bleeding has been given as a reason for seeking out CAM [34, 58, 63]. However, analysis of specific CAM use is scant, with use of these modalities being based on tradition or anecdotal evidence. Those women in our study were no more likely to use any CAM practitioner or CAM practice/product compared to non-sufferers. However women with heavy periods demonstrated a decreased likelihood of visiting a massage therapist or using yoga/meditation. Menorrhagia is likely to limit women’s daily activities due to discomfort and embarrassment and therefore these results are not unexpected [64].

Women with severe period pain in this cohort used limited CAM, being more likely to have used aromatherapy oils when dysmenorrhoea occurred with any sort of frequency and herbal medicines if the pain occurred often. There are a number of surveys into women’s choice of treatment for dysmenorrhoea and they have reported that over 50 % of women surveyed were satisfied with herbal medicine [32, 42, 58], vitamin/dietary supplements [32, 58] and acupuncture for ‘pelvic discomfort’ [58] however the latter two CAM were not significantly adopted by our cohort. A few recent clinical trials indicated aromatherapy may reduce dysmenorrhoea [65, 66] and traditional herbal medicines have established uterine spasmolytic properties and have also been effective in trials in reducing dysmenorrhoea [67, 68] lending support to the practice highlighted in our analysis.

For many women, both the regular occurrence of CPPD symptoms and lack of effective treatments offered, may explain sufferers frequent adoption of some CAM. That CAM practitioner visits are much less prevalent than CAM practice/product use leads to the conclusion that self-prescription in this sector is common and raises important issues of efficacy and safety in the absence of professional supervision. In addition, whilst CPPD categories have been largely compartmentalised in the literature, data from this survey indicates a great deal of crossover of CPPD symptoms within this age group. The overall prevalence of CPPD is 56.8 % in this cohort emphasising the significance of CPPD as a health issue amongst women aged 34 – 39 years and indeed this level is likely to be understated as only severe levels of dysmenorrhoea were recorded.

The limitations of our study are first the retrospective recording of both CAM use and CPPD symptoms which are therefore subject to recall bias. Second, due to the self-perceived nature of the CPPD symptoms examined and the lack of a clear definition to categorise them, subjective reporting makes data comparisons more difficult. The large sample size and otherwise representative nature of this cohort of 34–39 year old women, as well as the specific enquiry into those CAM commonly used in Australia does however provide valuable insights, especially for health providers, into the extent and preferential use of CAM for specific CPPD symptoms.

## Conclusion

This analysis has confirmed the high levels of CPPD symptoms and CAM use amongst women in this age group and provides the first detailed insight into the differential adoption of different individual CAM practitioners and practices/products across CPPD symptoms. Whilst women with PMS and severe dysmenorrhoea are using CAM, those with heavy and irregular bleeding may be unaware of existing CAM options. However, more extensive investigation is required to ascertain how effective and safe CAM use is in these circumstances, what is motivating their usage and how well informed all relevant health practitioners, as well as women with CPPD, are regarding the use of appropriate CAM.

## Ethics approval and consent to participate

Ethical approval for the ALSWH was gained from the Human Ethics Committees at the University of Queensland and University of Newcastle. The study participants provided written consent.

## Consent for publication

Not applicable.

## Availability of data and materials

The dataset supporting the findings of this article is available in the Australian Longitudinal Study on Women’s health website http://www.alswh.org.au/for-researchers. There is no restriction to its use by non-academics.

## Abbreviations

95 % CI: 95 % Confidence Interval.
ALSWH: Australian Longitudinal Study on Women’s Health
CAM: complementary and alternative medicine
CPPD: cyclic perimenstrual pain and discomfort
OR: odds ratio
PMS: premenstrual syndrome
